# Analysis of Determinants of Food Preferences in a Polish Population-Based Sample of Primary School Adolescents: Diet and Activity of Youth during COVID-19 (DAY-19) Study

**DOI:** 10.3390/nu15112504

**Published:** 2023-05-28

**Authors:** Aleksandra Kołota, Dominika Głąbska

**Affiliations:** Department of Dietetics, Institute of Human Nutrition Sciences, Warsaw University of Life Sciences (SGGW-WULS), 159c Nowoursynowska Street, 02-776 Warsaw, Poland; dominika_glabska@sggw.edu.pl

**Keywords:** diet, nutrition, food preferences, Food Preference Questionnaire (FPQ), children, adolescents, primary school, COVID-19 pandemic

## Abstract

For the choices of food products, food preferences are crucial, as they influence the intake of nutrients and the resultant quality of diet, but in Poland, no studies of food preferences were conducted during the COVID-19 pandemic on a population of young adolescents. The aim of this study was to analyze the determinants of food preferences in a Polish population-based sample of primary school adolescents as part of the Diet and Activity of Youth during COVID-19 (DAY-19) Study. The DAY-19 Study focused on a national sample of a population of primary school adolescents who were recruited based on cluster sampling of participants from counties and schools, yielding a sample of 5039 individuals. Their food preferences were assessed using the Food Preference Questionnaire (FPQ), and they were compared in subgroups stratified by (1) gender: male and female; (2) age: younger (10–13 years) and older (14–16 years); (3) place of residence: urban and rural; (4) Body Mass Index (BMI): underweight, normal body weight, and overweight/obese (assessed based on Polish growth reference values); and (5) physical activity level: low and moderate (assessed using the International Physical Activity Questionnaire for children aged 10–13 (IPAQ-C) and adolescents aged 14–16 (IPAQ-A)). In the population of adolescents, no statistically significant differences in food preferences between subgroups stratified by gender were observed (*p* > 0.05). For boys, none of the studied factors (age, place of residence, BMI, physical activity level) was statistically significant determinant of food preferences (*p* < 0.05), while for girls, all of them were statistically significant determinants (*p* > 0.05). All the assessed factors (age, place of residence, BMI, physical activity level) in girls were associated with preferences for snacks, and older girls, those from a rural environment, those who were underweight and overweight/obese, as well as those having a low physical activity level declared a higher preference for snacks than younger ones (*p* = 0.0429), those from an urban environment (*p* = 0.0484), those of a normal body weight (*p* = 0.0091), and those having a moderate physical activity level (*p* = 0.0083). Similarly, girls from rural environments declared a higher preference for starches than those from urban environments (*p* = 0.0103), and girls having a low physical activity level declared a higher preference for fruit than those having a moderate physical activity level (*p* = 0.0376). Taking this into account, the population of girls, in particular, needs dedicated educational actions to support proper nutritional habits. Additionally, older age, living in a rural environment, being underweight and overweight/obese, and having a low physical activity level may be indicated as factors predisposing one to food preferences potentially promoting unhealthy dietary habits.

## 1. Introduction

Adolescence is a period of intensive physical, mental, and social development, and health behaviors developed during this period, including nutritional behaviors, are commonly maintained throughout one’s life [1,2]. Taking this into account, it is believed that adolescents should be encouraged to implement health-promoting nutritional behaviors, which may support a lifelong, properly balanced diet and health [3]. However, for the choices of food products, food preferences are crucial, as they influence the intake of nutrients and the resultant quality of diet [4]. Food preferences are defined as attitudes that are expressed toward food products associated with how much people like or dislike specific foods, which are persistent from early childhood to later in life [5]. The results of studies conducted on populations of children and adolescents indicate numerous factors that may shape food preferences, including gender [6,7,8], age [4,9], environmental factors [10], weight [11], and physical activity level [12].

Food preferences are developed starting in early childhood, mainly influenced by the food practices of parents, but also by the environment [5]. However, in the case of adolescents, additional determinants must be considered, including the influence of peers, nutritional knowledge, familiarity with food products and dishes, and curiosity [13]. Moreover, food preferences may be changed throughout one’s life, influenced by individual determinants [4], but generally, the broadening of food preferences is observed, caused by social influence [14].

While children are becoming adolescents, they are gaining more independence from their parents, including, among other things, the possibility to make their own food choices [14]. Among various determinants of food choices, there are those associated with a product, such as its availability, attractiveness, convenience, or health-promoting values, and those associated with the consumer, such as hunger, mood, financial resources, or the image of one’s own body [15]. For adolescents, especially important determinants are associated with media and advertising, which are indicated to influence choices of specific food products [5].

During the COVID-19 pandemic, due to forced social isolation, adolescents were spending more time using electronic devices and being exposed to media and advertising [16]. As a result, the role of media in shaping food product choices and preferences may have increased during this period, which is detrimental mainly for food products with no significant health-promoting values, including processed foods being promoted by celebrities and influencers, which may consequently have negative impacts on health [17]. The data obtained so far indicate that during the COVID-19 pandemic, the changes in the nutritional behaviors of children and adolescents were both positive (decreased consumption of fast foods) and negative (decreased consumption of fruit and vegetables, increased consumption of sweets and snacks) [18]. At the same time, the problem of excessive body mass of adolescents during COVID-19 became even more serious than before [19], which may have also resulted from changes in food habits. Such changes were influenced by various factors, including anxiety, changing lifestyle, and limited possibilities of socializing during lockdowns [20].

In Poland during the COVID-19 pandemic, a population-based study of food preferences of older adolescents aged 15–20 years was conducted, confirming the association between food preferences and food choice determinants [21], food approach and food avoidance traits [22], as well as food habits [23]. However, no studies on food preferences were conducted during the COVID-19 pandemic on a population of younger Polish adolescents. Taking this into account, the aim of this study was to analyze the determinants of food preferences in a Polish population-based sample of primary school adolescents in the Diet and Activity of Youth during COVID-19 (DAY-19) Study.

## 2. Materials and Methods

### 2.1. Design of Study

The study was conducted as part of the Diet and Activity of Youth during COVID-19 (DAY-19) Study, which focused on a population of primary school adolescents. The first phase (in June 2020) included the assessment of food habits [24], their changes [25], and their association with body mass change [26], which indicated differences in nutritional behaviors before and during the COVID-19 pandemic. The study presented here was conducted during the second phase of the DAY-19 Study, from March to May 2021, and was approved by the Ethics Committee of the Central Clinical Hospital of the Ministry of Interior and Administration in Warsaw (no. 2/2021). The study was conducted in agreement with the Declaration of Helsinki, and participants and their parents/legal guardians provided their informed consent to participate in the study.

### 2.2. Studied Population

The population-based sample of primary school students was recruited for the study based on the same procedure used in the first phase of the DAY-19 Study [24,25,26]. The cluster sampling of participants from counties and schools was conducted in two stages: random sampling of counties from all voivodeships in Poland (voivodeships are the main administrative units of Poland), and random sampling of primary schools from previously sampled counties. In the first stage, 10 counties were sampled from each of the 16 voivodeships, and in the second, 10 schools were sampled from each county. The principals of schools were asked about their willingness to participate in the study, and they received the study protocol with all the necessary details. Finally, 42 primary schools participated and provided the informed consent of students and their parents/legal guardians who wanted to participate; for students and their parents/legal guardians, participation was voluntary. The students who provided informed consent received a link to the electronic questionnaire of the study, using the Computer-Assisted Web Interview (CAWI) method to gather the necessary data.

Taking into account the primary school students to which the study was addressed, being in 5th to 8th grade, and the attributed age in the Polish education system, being 11–15 years, the age of the gathered sample was assumed as 10–16 years, as it was possible to also sample younger or older students at the invited schools, and a 1-year age difference was assumed as acceptable.

The following inclusion criteria were settled:

- Student attributed to a sampled county and sampled school;
- Student aged 10–16 years;
- Provided informed consent to participate;
- Provided informed consent of parent/legal guardian for participation.
- The following exclusion criteria were settled:
- Missing data in the completed questionnaire;
- Answers in the questionnaire interpreted to be unreliable.

**Figure 1:**
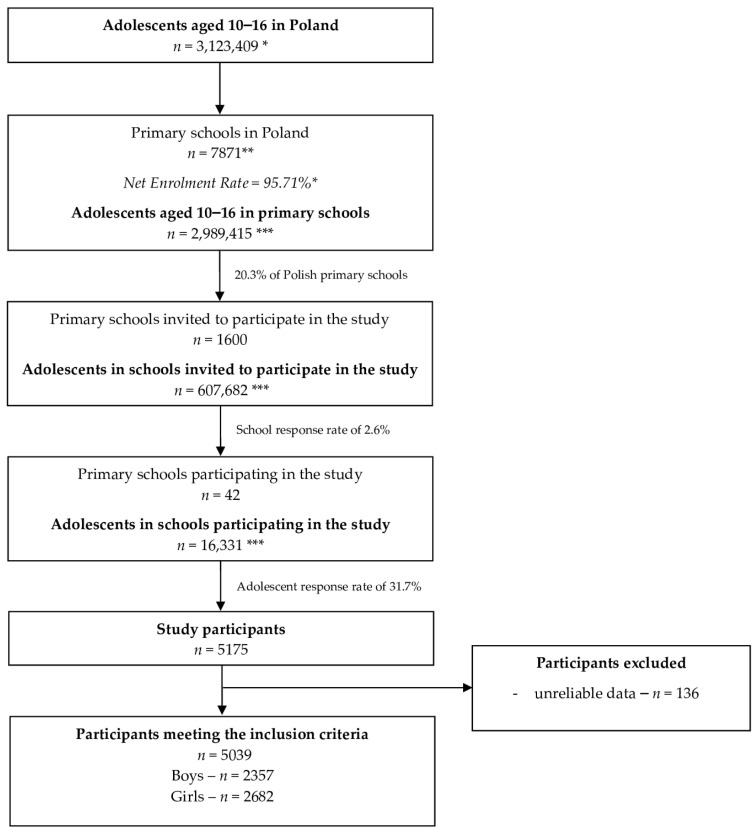
The sampling in the second phase of Diet and Activity of Youth during COVID-19 (DAY-19) DAY-19 Study. * Data from the Central Statistical Office of Poland (CSO) [27,28]; ** data from the Polish Ministry of National Education [29]; *** based on data from CSO.

The final sample gathered for the study was 5175 students, and the sampling procedure is presented in Figure 1.

### 2.3. Applied Questionnaire

The questionnaire applied in the study was anonymous, as it did not allow identifying participants, and no personal or sensitive information was gathered, but basic information about participants was obtained in order to verify the inclusion criteria.

The major element of the applied questionnaire was the Food Preference Questionnaire (FPQ), developed by Smith et al. [4], which was also used in previous studies on a population of adolescents during the COVID-19 pandemic [21,22,23]. The FPQ is a valid tool to gather information about the preferences for food products in groups of food products, which may be applied to populations of children and adolescents. It is self-reported, being the modified version of the previous tool, which gathered information about children’s preferences for food products through their parents [30]. In the FPQ, there are 62 food items included in the following groups of food products: vegetables (18 food items), fruit (7 food items), meat/fish (12 food items), dairy (10 food items), snacks (9 food items), and starches (6 food items). For each food item, the respondent is asked how much, on average, he/she likes it, and the following options may be chosen: dislike a lot; dislike a little; neither like nor dislike; like a little; like a lot. The question should be answered for each food item the respondent ever consumed or tried. Additionally, if a respondent does not know the listed food item, or does not remember consuming it, they may declare this, and the food item is then excluded from the analysis. The scores for each food item in a group of food products are recalculated into mean scores for the groups of food products [31].

At the same time, physical activity was assessed using the International Physical Activity Questionnaire for children and adolescents aged 10–13 (IPAQ-C) [32], and the International Physical Activity Questionnaire for adolescents aged 14–16 (IPAQ-A) [33]. Depending on age, the appropriate version was provided to assess various activities stratified in categories of activities, including those during school physical education classes, other classes, after classes, and during spare time, as well as during weekends. For each activity, the respondent was asked about number of times he/she performed a specific activity in the previous week, with the following options: not at all; 1–2 times; 3–4 times; 5–6 times; 7 times or more. The frequency was then recalculated into scores for each activity (from 1 for low activity to 5 for high activity), and the scores for each activity in the category of activities were recalculated into mean scores for the categories of activities [34]. In the conducted study, in order to stratify respondents by physical activity level, a score lower than 2 was attributed to a low level of physical activity, and a score of 2 or higher was attributed to a moderate level of physical activity (a score of 5 was not obtained in the studied population).

Moreover, the body mass was assessed, based on the Body Mass Index (BMI), calculated for body weight and height using the Quetelet equation [35]. The obtained value for each participant was converted into a centile of BMI, using the Polish growth reference values, based on gender and age [36], with dedicated software [37]. In order to stratify respondents by body mass, a BMI lower than the 5th centile is considered underweight, a BMI between the 5th and 85th centiles is considered normal body weight, and a BMI higher than the 85th centile is considered overweight/obesity, as commonly defined [38].

### 2.4. Statistical Analysis

Based on the basic information provided about participants, they were stratified by (1) gender: boys and girls; (2) age: younger (10–13 years) and older (14–16 years); (3) place of residence: rural and urban; (4) BMI: underweight, normal body weight and overweight/obese; and (5) physical activity level: low and moderate.

The distribution was verified using the Shapiro–Wilk test, and for nonparametric distribution, the Mann–Whitney U test and Kruskal–Wallis analysis of variance (ANOVA) were used. Additionally, the chi^2^ test was applied. The statistical analysis was conducted using Statistica version 13.3 (StatSoft Inc., Tulsa, OK, USA) and Statgraphics Plus for Windows 5.1 (Statgraphics Technologies Inc., The Plains, VA, USA), and *p* ≤ 0.05 was defined as statistically significant.

## 3. Results

The characteristics of the population of adolescents studied in the DAY-19 Study are presented in Table 1. It was found that the average BMI was significantly higher in boys than in girls (*p* < 0.0001), and more boys were overweight/obese than girls (*p* < 0.0001).

The food preferences assessed using FPQ in the population of adolescents studied in the DAY-19 Study are presented in Table 2. It was observed that the highest average preferences were for fruit (median of 4.29) and snacks (median of 4.20), while the lowest was for meat/fish (median of 2.64).

The food preferences assessed using FPQ in the population of adolescents studied in the DAY-19 Study, stratified by gender, are presented in Table 3. In the population of adolescents studied in the DAY-19 Study, no statistically significant differences in food preferences between subgroups stratified by gender were observed (*p* > 0.05).

The food preferences assessed using FPQ in the population of adolescents studied in the DAY-19 Study, stratified by age, are presented in Table 4. In the population of adolescents studied in the DAY-19 Study, no statistically significant differences in food preferences between subgroups stratified by age were observed (*p* > 0.05). In the population of boys, no statistically significant differences in food preferences between subgroups stratified by age were observed (*p* > 0.05). In the population of girls, no statistically significant differences in food preferences between subgroups stratified by age were observed (*p* > 0.05), except for snacks, as older girls declared higher preferences than younger ones (median of 4.30 vs. 4.20; *p* = 0.0429).

The food preferences assessed using FPQ in the population of adolescents studied in the DAY-19 Study, stratified by place of residence, are presented in Table 5. In the population of adolescents studied in the DAY-19 Study, no statistically significant differences in food preferences between subgroups stratified by place of residence were observed (*p* > 0.05), except for starches, as adolescents from rural environments declared higher preferences than those from urban environments (median of 3.83 vs. 3.75; *p* = 0.0040). In the population of boys, no statistically significant differences in food preferences between subgroups stratified by place of residence were observed (*p* > 0.05). In the population of girls, no statistically significant differences in food preferences between subgroups stratified by place of residence were observed (*p* > 0.05), except for snacks and starches, as girls from rural environments declared higher preferences for snacks (median of 4.30 vs. 4.20; *p* = 0.0484) and starches than those from urban environments (median of 4.00 vs. 3.75; *p* = 0.0103).

The food preferences assessed using FPQ in the population of adolescents studied in the DAY-19 Study, stratified by BMI, are presented in Table 6. In the population of adolescents studied in the DAY-19 Study, no statistically significant differences in food preferences between subgroups stratified by BMI were observed (*p* > 0.05). In the population of boys, no statistically significant differences in food preferences between subgroups stratified by BMI were observed (*p* > 0.05). In the population of girls, no statistically significant differences in food preferences between subgroups stratified by BMI were observed (*p* > 0.05), except for snacks, as girls of a normal body weight declared lower preferences than those who were underweight and overweight/obese (median of 4.20 vs. 4.30; *p* = 0.0091).

The food preferences assessed using FPQ in the population of adolescents studied in the DAY-19 Study, stratified by physical activity level, are presented in Table 7. In the population of adolescents studied in the DAY-19 Study, no statistically significant differences in food preferences between subgroups stratified by physical activity level were observed (*p* > 0.05), except for fruit and snacks, as adolescents having a low physical activity level declared higher preferences for fruit (median of 4.29 vs. 4.28; *p* = 0.0481) and snacks than those having a moderate physical activity level (median of 4.30 vs. 4.20; *p* = 0.0485). In the population of boys, no statistically significant differences in food preferences between subgroups stratified by physical activity level were observed (*p* > 0.05). In the population of girls, no statistically significant differences in food preferences between subgroups stratified by physical activity level were observed (*p* > 0.05), except for fruit and snacks, as girls having a low physical activity level declared higher preferences for fruit (median of 4.29 vs. 4.28; *p* = 0.0376) and snacks than those having a moderate physical activity level (median of 4.30 vs. 4.20; *p* = 0.0083).

## 4. Discussion

In the studied Polish population-based sample of primary school adolescents aged 10–16 years, the highest average preference was observed for fruit and snacks, which may have been expected for this population group. In the study by Cooke and Wardle [9] on a population of children, the highest preference was declared for sweet and fatty products. Such preferences may result in not following a properly balanced diet, as a sweet flavor preference is associated with a tendency to consume sweet products [39]. Moreover, the high preference for sweet and salty flavors is typical for adolescents, which commonly causes high consumption of sweets and snacks, resulting in a lower-quality diet [5]. A problem arises as adolescents often follow their preferences, even if they know nutritional recommendations; thus, unhealthy but preferred food products may become an important part of their diet [40].

In the presented study, gender was not statistically significant as a determinant of food preferences. Such results may be surprising, as in some studies, an association between gender and food preferences was observed. In the study by Caine-Bish and Scheule [41], boys had a higher preference for meat and fish, and girls had a higher preference for fruit and vegetables. Similarly, in the study by Cooke and Wardle [9], boys had a higher preference for fatty, sweet dishes, as well as meat and eggs, while girls had a higher preference for fruit and vegetables. However, it must be indicated that such declared differences may have resulted from a greater interest of girls in proper nutrition [13], as a consequence of a need to improve their body image by reducing body mass, typical for female respondents [42]. The lack of such differences in the study presented here may be due to various reasons, mainly the novel body positivity trend associated with emphasizing the beauty of each body, independently from body mass [43]. This trend supports girls and women in following their own preferences for food products instead of constantly dieting, but it must be indicated that this trend is sometimes interpreted as dangerous, as it is perceived by some social media users as a potential promotion of excessive body mass [44]. Moreover, the presented study was conducted during the period of the COVID-19 pandemic, which may have changed food-related priorities due to limited social contact, as it was indicated that adults during this period changed their approach to food [45]. The other problem may result from the level of stress experienced during the COVID-19 pandemic, as adolescents are especially vulnerable to prolonged stress [46]. Finally, the presented study was conducted on a group of diverse ages (from 10 to 16 years), and since body size dissatisfaction appears in girls during maturation [47], this may have not been a factor for the youngest respondents in the study.

Moreover, in the presented study, for boys, none of the studied factors (age, place of residence, BMI, physical activity level) were statistically significant determinants of food preferences, while for girls, all of them were statistically significant determinants. This difference may result from the varied approaches of parents and caregivers that shape food preferences and nutritional habits in children. As indicated by Scaglioni et al. [48] in their review, the nutritional habits of children are influenced by their parents offering them specific amounts of food and specific food products, and parents may have different approaches depending on the gender of their child; e.g., mothers more often give boys, rather than girls, food that is of a high energy value but unhealthy [49]. Such an approach may result in less self-regulation of eating behaviors (self-control during consumption) among boys than girls, which is commonly stated [50]. As a result, it may be indicated that in the studied group, there were no important determinants of declared food preferences in boys, as it may be supposed that they consume foods that they desire and that their preferences are not as influenced by external factors as observed for girls. Thus, no specific target groups of boys are indicated as prone to food preferences potentially promoting unhealthy dietary habits.

Meanwhile, target groups of girls may be identified, related to older age, living in a rural environment, being underweight and overweight/obese, and having a low physical activity level, as for all these groups, higher preferences for food products potentially promoting unhealthy dietary habits were observed.

For the age factor, it must be indicated that for younger children, parents and caregivers are crucial in shaping their dietary behaviors, whereas older children, including adolescents, want to present their independence in various ways, including in their own food choices [40]. During the COVID-19 pandemic, numerous changes in eating habits were observed, associated with increased consumption, especially of unhealthy food products [51]. So, it may be supposed that more independence in adolescents may be associated with following one’s food preferences, which in the presented study were less beneficial for this age group, resulting in unhealthy dietary habits. This corresponds with the results of studies indicating body mass gain in adolescents during the COVID-19 pandemic [52].

For the environment factor, in other Polish studies, the nutritional habits of adolescents from rural and urban environments differed; those among adolescents from rural environments were less beneficial [53], which corresponds to the less beneficial preferences of adolescents from rural environments observed in the presented study. Similarly, a Chinese study indicated changes in the food preferences of adolescents from urban and rural environments, being beneficial for adolescents from urban environments but not rural environments [54]. This indicates that the observed problem is universal and it contradicts the common assumption that inhabitants of rural areas have a more natural diet and that those in urban areas consume more highly processed foods [55]. This results from the dynamic development of rural areas and the urbanization of dietary habits, whereas in urban areas, people instead seek unprocessed foods, which are becoming of great value to them [56]. 

For the body mass factor, in the presented study, respondents of a normal body mass were characterized by more healthy food preferences, which may in fact have been the reason for their normal body mass. This is corroborated by the results of a Chinese study indicating that specific food preferences may be an important determinant of body mass [57], which suggests that food preferences influence body mass, rather than that body mass influences food preferences. However, some authors suggest that not only food preferences influence body mass, but that body mass also influences food preferences; this is suggested especially for excessive body mass individuals [58]. Such an association is indicated mainly for fatty products and it is suggested to result from multiple mechanisms, including seeking food rewards, as this mechanism may be stronger in excessive body mass individuals [59].

For the physical activity level, similarly to body mass, physical activity may influence food preferences (different preferences resulting from different lifestyles), and food preferences may influence physical activity (increased physical activity facilitated as a result of lower body mass). In the presented study, it was observed that a higher physical activity level was accompanied by more beneficial food preferences, which is in agreement with the results of other studies indicating that a low physical activity level is associated with a higher preference for food products of a high energy value [60]. Such an association results in better nutritional habits of physically active children than in those having a lower physical activity level [8], but it is also promoted by their greater nutritional knowledge [61]. Especially during the COVID-19 pandemic, when a reduction in physical activity was forced [62], more beneficial food preferences may have been a factor preventing body mass gain. This is also confirmed by the results of a study indicating that decreased physical activity and increased screen time are important determinants of improper nutritional habits [63].

Although the presented study revealed interesting associations, its strengths and limitations should also be presented. The major strength of the presented study is its large national population-based sample. Moreover, due to the limited number of studies conducted so far during the COVID-19 pandemic that assess the influence of food products, the role of the presented study may be significant. At the same time, the limitations of the study include the fact that self-reported food preferences may always be biased. Additionally, due to the COVID-19 pandemic, the study was conducted online. Finally, the study was conducted on a population of one country only, so no international perspective is presented.

## 5. Conclusions

In the studied Polish population-based sample of primary school adolescents aged 10–16 years, it was found that gender was not a determinant of food preferences. For boys, none of the studied factors (age, place of residence, BMI, physical activity level) was statistically significant determinant of food preferences, while for girls, all of them were statistically significant determinants. All the assessed factors (age, place of residence, BMI, physical activity level) were in girls associated with preferences for snacks, as older girls, those from rural environments, those who are underweight and overweight/obese, as well as those having a low physical activity level declared higher preferences for snacks than younger girls, those from urban environments, those of a normal body weight, and those having a moderate physical activity level. Similarly, girls from rural environments declared higher preferences for starches than those from urban environments, and girls having a low physical activity level declared higher preferences for fruit than those having a moderate physical activity level. Taking this into account, the population of girls, in particular, needs dedicated educational actions to support proper nutritional habits. Older age, living in a rural environment, being underweight and overweight/obese, as well as having a low physical activity level may be factors that predispose one to food preferences that potentially promote unhealthy dietary habits.

## Figures and Tables

**Table 1:** The characteristics of the population of adolescents studied in the Diet and Activity of Youth during COVID-19 (DAY-19) Study (*n* = 5039).

Parameter		Total (*n* = 5039)	Boys (*n* = 2357)	Girls (*n* = 2682)	*p* **
Age (years)	Mean ± SD	12.5 ± 1.5	12.5 ± 1.5	12.5 ± 1.4	0.0944
	Median (min–max)	12.0 (10–16) *	12.0 (10–16) *	13.0 (10–16) *	
Weight (kg)	Mean ± SD	49.7 ± 12.9	51.6 ± 14.5	47.9 ± 11.1	<0.0001
	Median (min–max)	48.0 (20.0–140.0) *	49.6 (20.0–140.0) *	47.0 (20.3–105.0) *	
High (cm)	Mean ± SD	159.4 ± 10.6	160.8 ± 12.2	158.2 ± 8.9	<0.0001
	Median (min–max)	160.0 (120.0–200.0) *	160.0 (120.0–200.0) *	159.0 (120.0–184.0) *	
BMI (kg/m^2^)	Mean ± SD	19.3 ± 3.6	19.7 ± 3.9	19.0 ± 3.4	<0.0001
	Median (min–max)	18.8 (10.0–50.3) *	19.0 (10.3–50.3) *	18.6 (10.0–37.7) *	
Underweight	*n* (%)	288 (5.7)	126 (5.4)	162 (6.0)	<0.0001
Normal body weight		3760 (74.6)	1677 (71.1)	2083 (77.7)	
Overweight/obesity		991 (19.7)	554 (23.5)	437 (16.3)	

* Nonparametric distribution (Shapiro–Wilk test; *p* ≤ 0.05); ** for comparison between boys and girls, the Mann–Whitney U test/chi^2^ test was applied.

**Table 2:** The food preferences assessed using the Food Preference Questionnaire (FPQ) in the population of adolescents studied in the Diet and Activity of Youth during COVID-19 (DAY-19) Study (*n* = 5039).

Group of Food Products	Total (*n* = 5039)			
	Mean ± SD	Median	25th Quartile	75th Quartile
Vegetable	3.12 ± 0.93	3.24 *	2.61	3.78
Fruit	4.02 ± 0.90	4.29 *	3.57	4.71
Meat/Fish	2.59 ± 0.93	2.64 *	2.00	3.27
Dairy	3.29 ± 0.84	3.44 *	2.78	3.89
Snacks	4.12 ± 0.74	4.20 *	3.70	4.70
Starches	3.78 ± 0.90	3.83 *	3.33	4.50

* Nonparametric distribution (Shapiro–Wilk test; *p* ≤ 0.05).

**Table 3:** The food preferences assessed using the Food Preference Questionnaire (FPQ) in the population of adolescents studied in the Diet and Activity of Youth during COVID-19 (DAY-19) Study (*n* = 5039), stratified by gender.

Group of Food Products	Boys (*n* = 2357)		Girls (*n* = 2682)		*p* **
	Mean ± SD	Median	Mean ± SD	Median	
Vegetable	3.15 ± 0.92	3.22 *	3.10 ± 0.94	3.23 *	0.1309
Fruit	4.06 ± 0.91	4.29 *	4.03 ± 0.90	4.29 *	0.0658
Meat/Fish	2.61 ± 0.94	2.64 *	2.58 ± 0.92	2.59 *	0.2091
Dairy	3.28 ± 0.84	3.44 *	3.29 ± 0.83	3.44 *	0.6449
Snacks	4.12 ± 0.75	4.20 *	4.12 ± 0.73	4.20 *	0.4555
Starches	3.78 ± 0.89	3.83 *	3.78 ± 0.91	4.00 *	0.8519

* Nonparametric distribution (Shapiro–Wilk test; *p* ≤ 0.05); ** for comparison, Mann–Whitney U test applied.

**Table 4:** The food preferences assessed using the Food Preference Questionnaire (FPQ) in the population of adolescents studied in the Diet and Activity of Youth during COVID-19 (DAY-19) Study (*n* = 5039), stratified by age.

Group of Food Products	Mean ± SD	Median	Mean ± SD	Median	
Total (*n* = 5039)	Younger (*n* = 3673)		Older (*n* = 1366)		*p* **
Vegetable	3.15 ± 0.92	3.28 *	3.14 ± 0.93	3.28 *	0.8657
Fruit	4.04 ± 0.91	4.29 *	4.06 ± 0.91	4.28 *	0.3067
Meat/Fish	2.61 ± 0.93	2.64 *	2.59 ± 0.94	2.64 *	0.4186
Dairy	3.29 ± 0.84	3.33 *	3.28 ± 0.84	3.33 *	0.8569
Snacks	4.22 ± 0.75	4.20 *	4.13 ± 0.74	4.20 *	0.3393
Starches	3.78 ± 0.90	3.83 *	3.77 ± 0.89	3.83 *	0.7134
Boys (*n* = 2357)	Younger (*n* = 1732)		Older (*n* = 625)		*p* **
Vegetable	3.14 ± 0.93	3.22 *	3.16 ± 0.88	3.22 *	0.7923
Fruit	4.06 ± 0.91	4.29 *	4.06 ± 0.88	4.29 *	0.7417
Meat/Fish	2.59 ± 0.94	2.64 *	2.63 ± 0.93	2.73 *	0.4819
Dairy	3.28 ± 0.86	3.44 *	3.28 ± 0.79	3.33 *	0.4319
Snacks	4.13 ± 0.76	4.30 *	4.10 ± 0.74	4.20 *	0.2055
Starches	3.76 ± 0.91	3.83 *	3.84 ± 0.84	4.00 *	0.1726
Girls (*n* = 2682)	Younger (*n* = 1941)		Older (*n* = 741)		*p* **
Vegetable	3.09 ± 0.94	3.23 *	3.10 ± 0.94	3.23 *	0.8803
Fruit	4.02 ± 0.90	4.29 *	4.05 ± 0.89	4.29 *	0.3355
Meat/Fish	2.58 ± 0.93	2.64 *	2.57 ± 0.89	2.54 *	0.4331
Dairy	3.29 ± 0.84	3.44 *	3.29 ± 0.82	3.44 *	0.8191
Snacks	4.11 ± 0.73	4.20 *	4.16 ± 0.73	4.30 *	0.0429
Starches	3.77 ± 0.92	3.83 *	3.79 ± 0.89	4.00 *	0.6447

* Nonparametric distribution (Shapiro–Wilk test; *p* ≤ 0.05); ** for comparison Mann–Whitney U test applied.

**Table 5:** The food preferences assessed using the Food Preference Questionnaire (FPQ) in the population of adolescents studied in the Diet and Activity of Youth during COVID-19 (DAY-19) Study (*n* = 5039), stratified by place of residence.

Group of Food Products	Mean ± SD	Median	Mean ± SD	Median	
Total (*n* = 5039)	Rural (*n* = 851)		Urban (*n* = 4188)		*p* **
Vegetable	3.11 ± 0.94	3.17 *	3.15 ± 0.91	3.28 *	0.3329
Fruit	4.04 ± 0.92	4.29 *	4.04 ± 0.90	4.29 *	0.6972
Meat/Fish	2.59 ± 0.94	2.64 *	2.59 ± 0.94	2.64 *	0.8794
Dairy	3.26 ± 0.86	3.33 *	3.29 ± 0.84	3.44 *	0.5311
Snacks	4.14 ± 0.75	4.30 *	4.12 ± 0.74	4.20 *	0.2989
Starches	3.77 ± 0.89	3.83 *	3.64 ± 0.99	3.75 *	0.0040
Boys (*n* = 2357)	Rural (*n* = 351)		Urban (*n* = 2006)		*p* **
Vegetable	3.11 ± 0.95	3.17 *	3.15 ± 0.93	3.28 *	0.3807
Fruit	4.06 ± 0.89	4.29 *	4.06 ± 0.91	4.29 *	0.8335
Meat/Fish	2.62 ± 0.95	2.72 *	2.60 ± 0.94	2.64 *	0.6349
Dairy	3.24 ± 0.89	3.33 *	3.29 ± 0.85	3.44 *	0.4355
Snacks	4.09 ± 0.76	4.20 *	4.13 ± 0.75	4.20 *	0.5349
Starches	3.75 ± 0.85	3.83 *	3.64 ± 0.99	3.75 *	0.1645
Girls (*n* = 2682)	Rural (*n* = 500)		Urban (*n* = 2182)		*p* **
Vegetable	3.12 ± 0.94	3.22 *	3.14 ± 0.89	3.28 *	0.6236
Fruit	4.03 ± 0.94	4.29 *	4.03 ± 0.89	4.29 *	0.4417
Meat/Fish	2.56 ± 0.93	2.55 *	2.59 ± 0.93	2.64 *	0.5754
Dairy	3.27 ± 0.84	3.39 *	3.28 ± 0.84	3.39 *	0.8687
Snacks	4.16 ± 0.75	4.30 *	4.11 ± 0.73	4.20 *	0.0484
Starches	3.78 ± 0.91	4.00 *	3.65 ± 0.99	3.75 *	0.0103

* Nonparametric distribution (Shapiro–Wilk test; *p* ≤ 0.05); ** for comparison, Mann–Whitney U test was applied.

**Table 6:** The food preferences assessed using the Food Preference Questionnaire (FPQ) in the population of adolescents studied in the Diet and Activity of Youth during COVID-19 (DAY-19) Study (*n* = 5039), stratified by Body Mass Index (BMI).

Group of Food Products	Mean ± SD	Median	Mean ± SD	Median	Mean ± SD	Median	
Total (*n* = 5039)	Underweight (*n* = 288)		Normal Body Weight (*n* = 3760)		Overweight/Obese (*n* = 991)		*p* **
Vegetable	3.08 ± 0.97	3.17 *	3.16 ± 0.91	3.28 *	3.16 ± 0.90	3.28 *	0.4875
Fruit	4.05 ± 0.92	4.29 *	4.05 ± 0.90	4.29 *	4.02 ± 0.92	4.29 *	0.7841
Meat/Fish	2.63 ± 0.95	2.64 *	2.60 ± 0.94	2.64 *	2.56 ± 0.90	2.55 *	0.2739
Dairy	3.28 ± 0.88	3.44 *	3.29 ± 0.84	3.44 *	3.29 ± 0.80	3.33 *	0.9565
Snacks	4.13 ± 0.75	4.30 *	4.12 ± 0.74	4.20 *	4.13 ± 0.73	4.20 *	0.7492
Starches	3.73 ± 0.89	3.83 *	3.78 ± 0.90	3.83 *	3.79 ± 0.89	4.00 *	0.3729
Boys (*n* = 2357)	Underweight (*n* = 288)		Normal body weight (*n* = 3760)		Overweight/obese (*n* = 991)		*p* **
Vegetable	2.99 ± 1.05	3.11 *	3.16 ± 0.913	3.28 *	3.17 ± 0.90	3.31 *	0.2639
Fruit	3.87 ± 1.07	4.07 *	4.07 ± 0.91	4.29 *	4.01 ± 0.95	4.29 *	0.1107
Meat/Fish	2.57 ± 0.99	2.64 *	2.59 ± 0.94	2.64 *	2.52 ± 0.85	2.55 *	0.2312
Dairy	3.23 ± 0.98	3.44 *	3.29 ± 0.83	3.44 *	3.26 ± 0.79	3.33 *	0.5551
Snacks	4.04 ± 0.80	4.20 *	4.13 ± 0.75	4.20 *	4.09 ± 0.73	4.20 *	0.1604
Starches	3.64 ± 0.87	3.83 *	3.78 ± 0.91	3.83 *	3.76 ± 0.92	4.00 *	0.1261
Girls (*n* = 2682)	Underweight (*n* = 162)		Normal body weight (*n* = 2083)		Overweight/obese (*n* = 437)		*p* **
Vegetable	3.15 ± 0.89	3.19 *	3.15 ± 0.89	3.28 *	3.15 ± 0.91	3.28 *	0.9832
Fruit	4.19 ± 0.75	4.29 *	4.03 ± 0.88	4.29 *	4.04 ± 0.89	4.29 *	0.1041
Meat/Fish	2.68 ± 0.92	2.73 *	2.61 ± 0.93	2.64 *	2.62 ± 0.94	2.64 *	0.6446
Dairy	3.32 ± 0.79	3.44 *	3.29 ± 0.84	3.44 *	3.24 ± 0.82	3.44 *	0.6805
Snacks	4.19 ± 0.69	4.30 *^a^	4.11 ± 0.73	4.20 *^b^	4.19 ± 0.73	4.30 *^a^	0.0091
Starches	3.80 ± 0.88	4.00 *	3.78 ± 0.90	3.83 *	3.84 ± 0.87	4.00 *	0.5048

* Nonparametric distribution (Shapiro–Wilk test; *p* ≤ 0.05); ** for comparison, Kruskal–Wallis ANOVA was applied.

**Table 7:** The food preferences assessed using the Food Preference Questionnaire (FPQ) in the population of adolescents studied in the Diet and Activity of Youth during COVID-19 (DAY-19) Study (*n* = 5039), stratified by physical activity level.

Total (*n* = 5039)	Low (*n* = 962)		Moderate (*n* = 4077)		
Group of Food Products	Mean ± SD	Median	Mean ± SD	Median	*p* **
Vegetable	3.17 ± 0.93	3.28 *	3.15 ± 0.90	3.28 *	0.4308
Fruit	4.09 ± 0.89	4.29 *	4.03 ± 0.91	4.28 *	0.0481
Meat/Fish	2.59 ± 0.95	2.64 *	2.60 ± 0.92	2.64 *	0.9979
Dairy	3.30 ± 0.86	3.44 *	3.28 ± 0.83	3.33 *	0.1702
Snacks	4.16 ± 0.74	4.30 *	4.11 ± 0.74	4.20 *	0.0458
Starches	3.80 ± 0.89	4.00 *	3.78 ± 0.90	3.83 *	0.4865
Boys (*n* = 2357)	Low (*n* = 486)		Moderate (*n* = 1871)		*p* **
Vegetable	3.13 ± 0.97	3.22 *	3.14 ± 0.90	3.22 *	0.7935
Fruit	4.06 ± 0.94	4.28 *	4.05 ± 0.89	4.28 *	0.5120
Meat/Fish	2.61 ± 0.96	2.61 *	2.62 ± 0.94	2.64 *	0.8979
Dairy	3.26 ± 0.89	3.26 *	3.28 ± 0.83	3.44 *	0.9932
Snacks	4.11 ± 0.79	4.30 *	4.13 ± 0.74	4.20 *	0.8581
Starches	3.79 ± 0.87	4.00 *	3.78 ± 0.89	3.83 *	0.8980
Girls (*n* = 2682)	Low (*n* = 476)		Moderate (*n* = 2206)		*p* **
Vegetable	3.21 ± 0.88	3.33 *	3.16 ± 0.91	3.28 *	0.3867
Fruit	4.12 ± 0.82	4.29 *	4.01 ± 0.91	4.28 *	0.0376
Meat/Fish	2.56 ± 0.93	2.55 *	2.57 ± 0.91	2.75 *	0.8130
Dairy	3.36 ± 0.82	3.44 *	3.28 ± 0.83	3.33 *	0.0523
Snacks	4.20 ± 0.68	4.30 *	4.10 ± 0.74	4.20 *	0.0083
Starches	3.81 ± 0.91	4.00 *	3.77 ± 0.92	3.92 *	0.3606

* Nonparametric distribution (Shapiro–Wilk test; *p* ≤ 0.05); ** for comparison, Mann–Whitney U test was applied.

## Data Availability

Data available on request due to ethical restrictions.
